# GLOBE Mosquito Habitat Mapper Citizen Science Data 2017–2020

**DOI:** 10.1029/2021GH000436

**Published:** 2021-10-01

**Authors:** Russanne Low, Rebecca Boger, Peder Nelson, Matteo Kimura

**Affiliations:** ^1^ Institute for Global Environmental Strategies Arlington VA USA; ^2^ Brooklyn College Earth and Environmental Sciences Brooklyn NY USA; ^3^ Oregon State University College of Earth Ocean, and Atmospheric Sciences Corvallis OR USA; ^4^ NASA SEES Summer Internship Program Participant University of Texas Austin TX USA

**Keywords:** citizen science, data quality, geohealth, mobile app, mosquitoes

## Abstract

The GLOBE Program's GLOBE Observer application is a free citizen science mobile data collection and visualization tool compatible with iOS and Android operating systems. Citizen scientists armed with the app can report the mosquito larval habitats they identify using the GLOBE Mosquito Habitat Mapper tool. This data can complement the climate, weather, and land cover data obtained from satellite measurements by scientists who develop risk models for mosquito‐borne diseases. Public participation in mosquito surveillance research provides the opportunity to obtain the volume, velocity and variety of data needed to fight the threat of vector‐borne diseases, especially in under‐resourced communities with minimal to no municipal surveillance and mitigation services. GLOBE Mosquito Habitat Mappers document and describe potential and active mosquito larval habitats in and around their homes and communities. An easy‐to‐use pictorial interface enables users to geolocate and describe oviposition sites encountered, count and identify mosquito larvae, and when appropriate, eliminate the larval habitats. During Mosquito Habitat Mapper's first 3 years of use, over 24,000 data observations have been reported throughout the world. This technical report summarizes GLOBE Mosquito Habitat Mapper data reported by GLOBE citizen scientists from three regions: Africa, Asia and the Pacific Islands, and Latin America and the Caribbean. Localized mosquito larvae distribution patterns were examined by comparing data collected in three cities in Senegal–Dakar, Touba, and Thilmakha. The Senegal data show habitat and genera differences among mosquitoes identified by citizen scientists in the cities and illustrates the potential of the app for community‐based surveillance and research.

## Key Points

- GLOBE Observer is The GLOBE Program's mobile app. The GLOBE Mosquito Habitat Mapper on this platform enables citizen scientists to identify, describe and report mosquito larvae and habitats
- GLOBE Mosquito Habitat Mappers not only collect data, but participate in source reduction and reduce risk of disease in their community
- GLOBE Mosquito Habitat Mapper data has practical applications in municipal surveillance as well as in mosquito vector borne disease research

## 1 Introduction

Citizen science is a form of public participation in scientific research where volunteer members of the public and research scientists collaborate to address real world problems (Bonney et al., 2009, 2014; Shirk et al., 2012). In the last decade, participatory research projects have grown in popularity with the public, with millions of people volunteering on projects every year (Bowser et al., 2020; Callaghan et al., 2019). Increasingly, scientists are using citizen science data in their research that spans biological conservation to mapping galaxies (McKinley et al., 2017; Shanley, 2020). Recently, the value of the volume, velocity, and variety of data created by non‐specialist volunteers using personal mobile devices has also attracted the attention of public health researchers (Katapally, 2020; Wang et al., 2019). When it comes to pandemics, whether COVID‐19 or mosquito‐vector borne disease, Katapally suggests that the tool we need to obtain critical data and to reinforce health‐fostering behaviors is “perhaps right in our pockets.”

In this paper we describe the data reported by citizen scientists using the GLOBE Mosquito Habitat Mapper tool in Africa, Asia and the Pacific Islands, and Latin America and the Caribbean during the first three years since its release on the GLOBE Observer mobile app. After describing the GLOBE Observer app and the GLOBE Mosquito Habitat Mapper tool, we present the raw data obtained from citizen scientists. Next, we describe the quality control steps employed in developing the data set used in this analysis. Finally, Mosquito Habitat Mapper data from three cities in Senegal are compared, to demonstrate the potential of high granuality data when described at the community level.

## 2 Materials and Methods

### 2.1 GLOBE Observer Mosquito Habitat Mapper App Description

The Global Learning and Observations to Benefit the Environment (GLOBE) Program is an international science and education program engaging students, teachers, community members and scientists in Earth system monitoring and research (www.globe.gov). Since its inception 25 years ago, GLOBE has supported authentic inquiry‐based science in K‐12 classrooms in 124 countries though a student environmental monitoring program that applies scientist‐developed research protocols and data reporting procedures. In 2016, The GLOBE Program launched the GLOBE Observer (GO) mobile application (app), expanding its mission to include the participation of citizen scientists at large, both broadening the reach of NASA science and increasing the spatial and temporal density of environmental observations reported to the database (Amos & Andersen, 2019).

Citizen scientists using the GO app provide in‐situ, ground reference data that can be used by scientists to analyze and interpret digital data derived by sensors on remote platforms. Terrestrial, hydrologic and atmospheric observations are collected and reported using four‐in app protocols: Clouds, Land Cover, Tree Height, and Mosquito Habitat Mapper. In the field, coincident observations using two or more protocols are encouraged because this provides citizen scientists with an opportunity to observe and document firsthand some of the complex Earth system interactions that are responsible for the changes we see taking place on our planet.

Internet connectivity is not required to make and record observations using the app: Data are stored in the mobile device until sufficient bandwidth is available for uploading data to the GLOBE database. GLOBE data are publicly available and accessible in a variety of ways. Data can be downloaded as KML or CSV files using the GLOBE Visualization Tool (GLOBE Vis), the Advanced Data Analysis Tool (ADAT) or through an Application Programming Interface (API) from the GLOBE website (https://www.globe.gov/globe-data). Descriptions of GLOBE data visualization and access procedures, metadata descriptions, and quality assurance practices are detailed in Amos and Andersen (2019) and GLOBE (2019).

The GLOBE Mosquito Habitat Mapper tool was released in May 2017 to provide in‐situ ground based observations to support scientific research using environmental data obtained from satellites. Scientists who develop risk models for mosquito borne diseases frequently employ remotely sensed data, but the mosquito vectors themselves obviously can't be seen from remote sensing platforms in space. Mosquitoes are arthropods whose immature forms are aquatic, and they respond sensitively to changes in environmental conditions such as precipitation, temperature and humidity. Along with weather and climate, land cover data are frequently analyzed by scientists who conduct mosquito vector borne disease research (see systematic review by Sallam et al., 2017). There is a pressing need for fine‐grained in‐situ data describing relationship of mosquito larval habitats to the land cover classes identified in satellite products (Lorenz et al., 2020) and thus in‐situ observations of mosquito larval habitats are needed, especially in regions where variable conditions are pronounced or where change is occurring rapidly. Citizen scientists, working in conjunction with local public health and mosquito control authorities, are situated to fill this data gap. To support community‐level engagement, the GO app enables groups to establish a team, a feature that enables data from a local mosquito surveillance group, campaign or event to be readily associated together and analyzed.

As a built‐in incentive for citizen scientists who collect data needed by the scientific community, their actions performed as part of the GLOBE Mosquito Habitat Mapper protocol simultaneously reduce the risk of mosquito vector borne disease in and around their homes and neighborhoods. By reporting and mitigating mosquito larval habitats, citizen scientists eliminate thousands of immature mosquitoes that otherwise would become biting adults and potential vectors of human disease.

By design, GLOBE Mosquito Habitat Mapper citizen scientists can participate in data collection at both casual and dedicated levels of engagement. To accommodate a citizen scientist's interest, time constraints, access to simple equipment, and/or ability, the app is designed to allow the volunteer to terminate their observation at each of the 4 steps in the app. This opens participation to individuals in a broad range of ages (13+) and skill levels, from novice to expert amateur to professional scientist. The steps in the GLOBE Mosquito Habitat Mapper protocol are described below.

Step 1: Mosquito larval habitat documentation. The first data collection step involves identifying and photo documenting potential mosquito larval habitats on the landscape. Users are prompted to look for standing water sources‐natural or artificial‐where an adult mosquito might lay her eggs. For example, users might find discarded items such as cans, bottles or old tires, or purposeful water storage containers, such as cisterns or rain barrels that serve as oviposition sites. Natural standing water sources include ponds, puddles, marshes and estuaries. GLOBE Observers classify the mosquito larval habitat they observe into one of 32 categories (including “other”) by making selections from images in a graphic interface. Next, they are instructed to document their observation with up to nine photos of the mosquito habitat and surrounding area. The app prompts them to report whether mosquito larvae are visible in the standing water source. A series of yes/no questions follow where users report whether they observe larvae, pupae, adults or eggs at their larval habitat: Reference images are provided for reference by novice users. Once the larval site has been documented, citizen scientists can either terminate observations or continue to the next step.

Step 2: Determine if immature mosquitoes are present in the habitat. Using a macropipette or mosquito dipper, citizen scientists are asked to take a sample of the water and enter separate counts for larvae and pupae observed. If no immature specimens are detected, users are instructed to enter “zero” as the larvae and/or pupae counts. If larvae are observed, users are prompted to photograph representative specimens, using the zoom feature of the camera in their mobile device. Interested citizen scientists are encouraged to obtain an inexpensive (less than $10 US) 60x macrolens that can be clipped onto their mobile camera lens to capture images of sufficient resolution to identify to genera. To support expert identification, voucher photos of larvae are stored at their original image resolution in the GLOBE database. Users are then given the option to terminate observations at this step or continue to specimen identification.

Step 3: Taxon identification. Users are prompted to capture a full body photo of their specimen, as well as a close‐up photo of the diagnostic features located on the terminal anal segments used for discriminating between taxa. Once a photo is taken, close‐up larva photo is displayed at the top of each step in the key for convenient comparison with diagnostic images.

Citizen scientists employ an in‐app pictorial dichotomous taxonomic key to determine whether their larva specimen belongs to one of three medically important genera found worldwide: *Aedes*, *Culex* or *Anopheles* (WHO, 2020). The app's information system collects user data at each step in the key, so that if users end their identification task before identifying the specimen to genus, the last step attempted is recorded. This feature provides a record of the precision of citizen scientist identifications (Lukyanenko et al., 2019). At several points in the identification process, citizen scientists have the option of selecting “I'm not sure,” a feature of the information system that reduces false reports and enhances data quality (Torre et al., 2019). If citizen scientists would like to identify different genera than the three targeted ones, they can use a local mosquito identification key and enter the genus or species into the app where they write comments.

Step 4: Source reduction. Regardless of the number of steps previously completed, all users end their observation by indicating if they were able to mitigate the mosquito larval habitat site by removing, dumping out, or covering a water container. This action, known within the mosquito control community as *source reduction*, is a is an important behavioral practice which has a measurable impact on the transmission of dengue, malaria and other diseases (Forsyth et al., 2020; Gu et al., 2006).

Once the observation is completed, the user is prompted to make a coincident observation using the Land Cover tool, also found on the GO app. Then the user is prompted to either upload their data to the GLOBE database, or alternatively, store the data in the device until an internet connection is available. Each citizen scientist is able to access a collection of their own data in app. Personally reported Land Cover observations can also be reviewed in. app via a map interface.

In the following section, we describe the data obtained using the GLOBE Mosquito Habitat Mapper for the three years since its release on the GO platform, from May 29, 2017 through May 28, 2020.

After describing the data as a whole, we compare three world regions and present a limited case study of data reported by citizen scientists from Senegal.

## 3 GLOBE Mosquito Habitat Mapper Data

### 3.1 Raw Data

**Figure 1:**
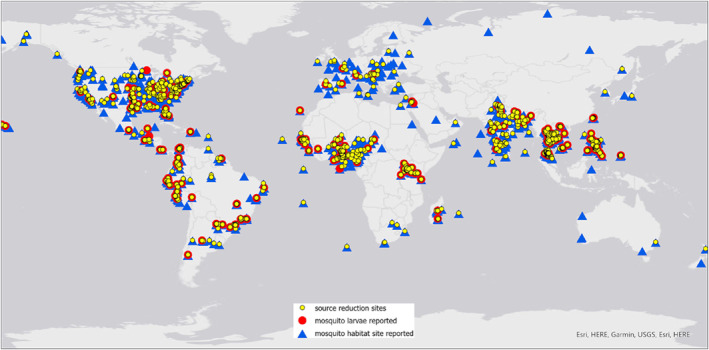
Geolocated GLOBE Mosquito Habitat Mapper data displayed using the GLOBE Visualization System, May 29, 2017 to May 28, 2020. All reported data, *n* = 24,983. Legend: *Triangles:* Locations of reported mosquito larval habitats, *n* = 8,335. *Red circles:* locations with reported mosquito genera, *n* = 1,336. *Yellow circles:* Locations where breeding sites were mitigated, *n* = 8,307. Not pictured: locations with accompanying photographic data, *n* = 6,915. Source: GLOBE Visualization System, August 1, 2020.

GLOBE Mosquito Habitat Mappers using the GLOBE Observer app are active in 73 countries and contributed more than 24K observations during the period of study, May 29, 2017 through May 28, 2020 (Figure 1). Reported observations have increased steadily until 2020: 2,153 observations were reported in 2017, followed by 6,726 observations in 2018, 13,328 observations in 2019, totaling more than 24K observations by May, 2020. Citizen scientists are able to decide which steps they want to complete, so not all attributes identified in the protocol are reported in each record. In this study, a complete record is defined in this study as a data submission including mosquito habitat description, larval count, and an attempt to identify specimens to genus. During the period of study, 5,138 complete records were uploaded by volunteers.

After downloading data using the GLOBE API, we automated data cleaning using Python scripts. The scripts, resulting data sets and analytical procedures are available at https://geospatial.strategies.org/pages/publication-data.

### 3.2 Removal of Records With Suspect Geolocation Data

Knowing the spatial accuracy of a data set is critical to determining its fitness of use for a specific research application (Chapman, 2005). Each record in the GLOBE database is identified by two sets of coordinates: The geolocation obtained using the receiver on the citizen scientist's mobile device, and the site's position within the Military Grid Reference System (MGRS). For naming and visualization purposes, GLOBE site IDs are associated with the latitude and longitude coordinate of the lower left corner of a 100 × 100 m grid cell aligned to the Military Grid Reference System (MGRS) (GLOBE, 2019).

We ran Python scripts to remove locations of insufficient precision: These included records with locations identified by whole numbers, rather than decimal degrees, as well as suspect and anomalous locations (such as in the ocean). “Measured locations” that were autopopulated by MGRS grid coordinates were obviously not measured and were also removed from the data set. This procedure removed 5,704 (23%) of the 24,983 records listed in the GLOBE Mosquito Habitat Mapper database, with 19,279 records remaining.

### 3.3 Identifying Suspected Training Events

When we imported the unfiltered GLOBE Mosquito Habitat Mapper data into ArcGIS Pro analysis software (ESRI, 2020), we identified cases where more than one record reported the same geographic coordinates and water sources. Visual inspection of larval photos associated with these records showed multiple cases where several citizen scientists submitted submitting photos of the same specimen: Identified either by the position of the larva, unique time‐limited observable features (such as patterns and coloration in the alimentary canal), or the same detritus in the suspending liquid, with similar geolocation, date, and time provenience. Realizing that many citizen scientists learn how to use the GLOBE Mosquito Habitat Mapper in workshops at local, regional, and national GLOBE training events, we explored methods to capture and remove suspected duplicate data obtained during training events.

The records were first sorted using the GLOBE Site ID location. We identified records that shared the same MGRS grid coordinates, the same day of submission, and the same water source. For this test, we sought to identify groups of data that exceeded 10 records sharing these characteristics and subjected those groups to visual inspection. Because we needed to manually review the photo records, we set the identification threshold for a group at >10, so that the analysis could be completed in the time allotted. The analysis identified 2,447 records that were postulated to be duplicate data uploaded during training events.

### 3.4 Data Description

The following section describes the cleaned datasets obtained from citizen scientists from the GLOBE regions of Africa, Asia and the Pacific, and Latin America and the Caribbean. These data are summarized in Table 1.

**Table 1:** Summary Table for GLOBE Observer Mosquito Habitat Mapper Data for the Africa, Asia and Pacific, and Latin America and Caribbean Regions, Reported by Protocol Step. Blue Column=Raw Counts. White Column=Raw Count as Percentage of Total

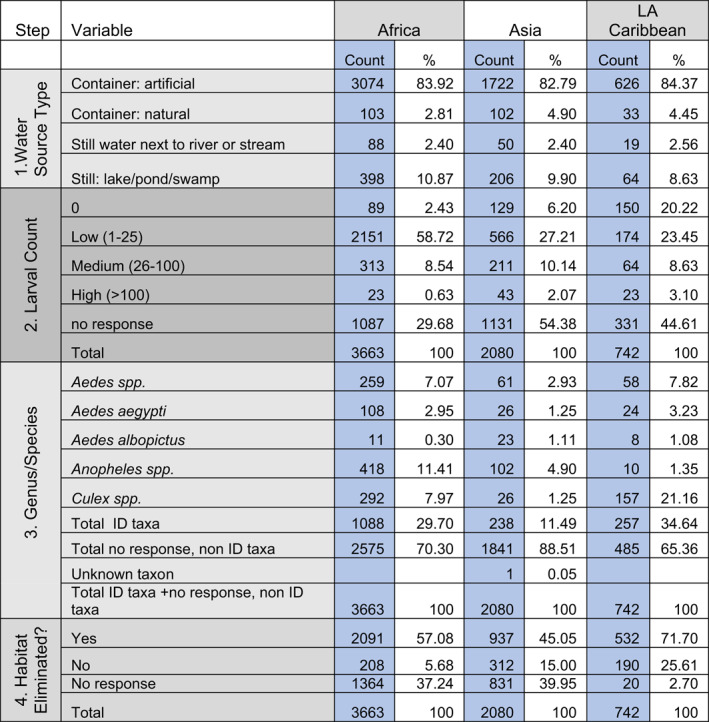

**1. Water source type.** In the app, the water source type is described by four broad categories of standing water. Open water habitats include permanent or semi‐permanent sources (such as a lake, pond, or swamp) and ephemeral, impounded still water puddle next to an actively flowing river or stream (still water next to river or stream). The other two categories of standing water include water found in artificial (manufactured) containers (such as jars, cisterns) or natural containers (such as treeholes or an empty coconut husk). Within these categories, citizen scientists used a pictorial key in the app to select from 32 different subcategories that refine the habitat characteristics.

The reported habitats were similar across all three regions. Over 80% of the records reported artificial container larval habitats. Ranging from 9% to 11%, lake/pond/river was the second most reported category of larval habitat. Because GLOBE Observer workshops have tended to stress the importance of identifying and mitigating container habitats, bias in reported observations is expected, and significance is attributed to the relative number of artificial containers versus natural water sources in sampling locations.

With respect to artificial (manufactured) containers, citizen scientists in the Latin America and Caribbean region reported far more jars and mosquito traps (intentionally set, scientific). In the Asia and Pacific region and Africa region, the most frequently reported larval habitats were cement, metal or plastic tanks. In the Asia and Pacific region, fountain/bird bath, dish/pot, and well/cistern were next in importance, as were dishes/pots and used tires in the African Region.

**2. Larvae count categories.** Actual larval counts for each sampling location are reported by citizen scientists. We summarized the counts into three categories: low (1–25), medium (26–100) and high (over 100). Of those mosquito larval habitats where citizen scientists conducted larvae counts, the majority reported between 1 and 25 specimens (low). Between 30% and 54% of submitted records did not include larvae counts.

**3. Genus/species identification.** The identification of larvae specimens using Mosquito Habitat Mapper's built‐in dichotomous taxonomic key requires a significant amount of time as well as access to additional inexpensive equipment. These include a water sampling tool, such as either a mosquito dipper or cup, or a macropipette (turkey baster), and a 60–100x clip‐on macrolens for use with the mobile device's built‐in camera.

Submitted records that included a specimen identification from the habitat were lowest among participants in the Asia and Pacific region (12%) followed by Africa (30%). Participants from the Latin America and Caribbean region provided a specimen identification 35% of the time. Differentiating *Aedes aegypti* or *Aedes* optically from other *Aedes* taxa requires careful observation and at least 100x magnification. Citizen scientists identified *Aedes aegypti* or *Aedes albopictus* specimens in less than 5% of the cases reported. Photos are submitted of the whole body and close‐ups of the abdomen with key identifying features. These photos serve as voucher specimens for validating identifications by citizen scientists. Development of image recognition software using artificial intelligence is underway, which will greatly assist in validating citizen scientist larval identifications by automating the process, thus reducing the time and labor involved in manual expert examinations of submitted photos (Muñoz et al., 2019).

**4. Habitat Eliminated?** Citizen scientists in the Latin America and Caribbean region responded to the question, “Did you dump out the water?” 96% of the time, with 72% of the responses affirmative. Slightly more than 60% of citizen scientists from the Asia and the Pacific and Africa regions answered the source reduction question for the site they identified, answering yes in 57% (Africa) and 47% (Asia and Pacific Islands) of the records. The differences can be partially attributed to the types of water sources, because it would be difficult to mitigate a swamp or cement tank without the use of chemicals, and a large percentage of observed larval habitats in Africa were cement, metal, or plastic tanks.

### 3.5 Senegal Case Study

**Figure 2:**
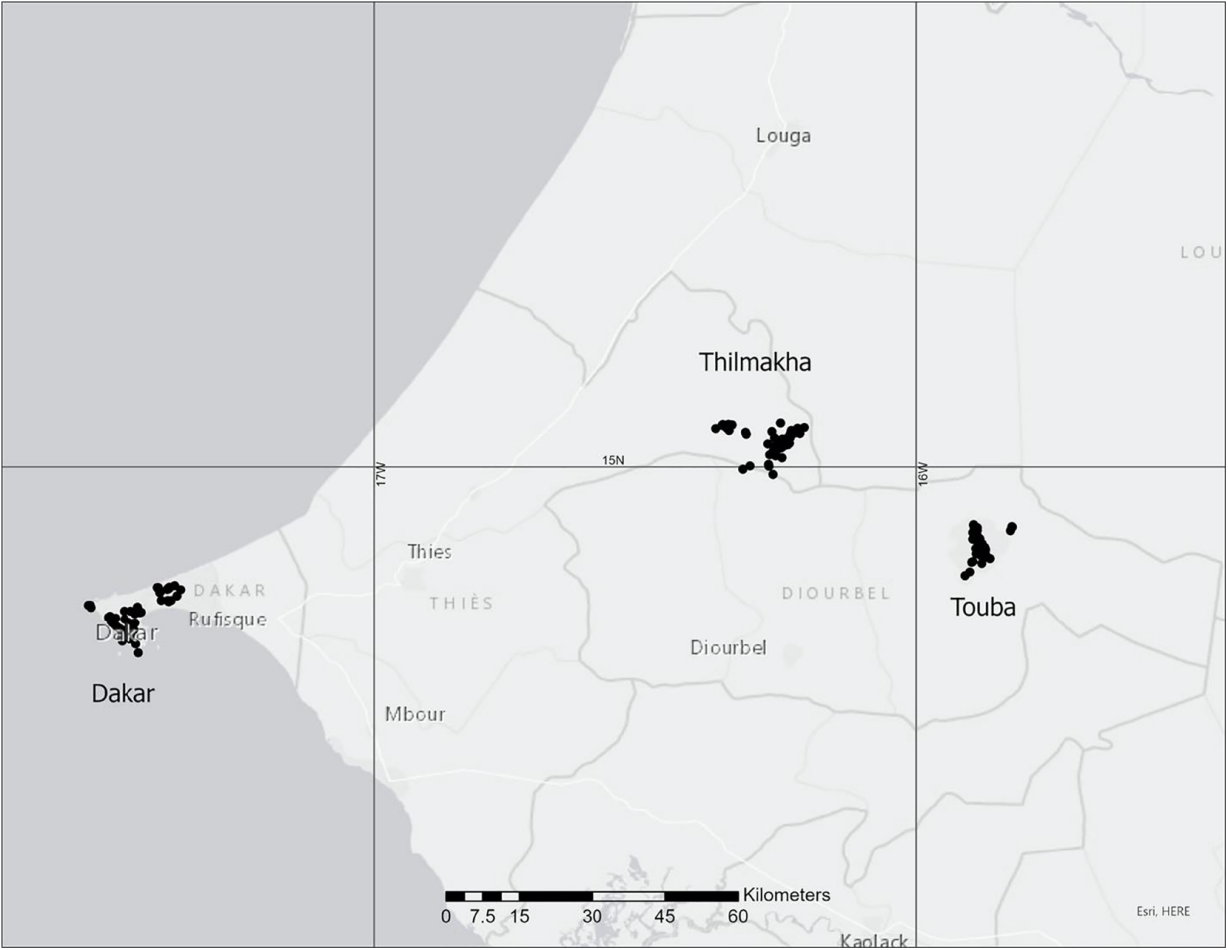
Center: Map showing clustered sampling locations in in each of the three locations. Dakar ‐ large urban, Thilmakha ‐ rural village, and Touba, town.

This section describes data reported from citizen scientists in Senegal, from October 31, 2018, the first data entry from this country, until May 31, 2020, the end of the period of this analysis. In this case study, data from three locations are examined: A large urban center (Dakar), a rural village (Thilmakha) and a town (Touba) (Figure 2). The discussion refers to data presented in Table 2.

**Table 2:** Citizen Science Data Reported From Dakar, Thilmakha and Touba, Senegal. Blue Column=Raw Counts. White Column=Raw Count as Percentage of Total

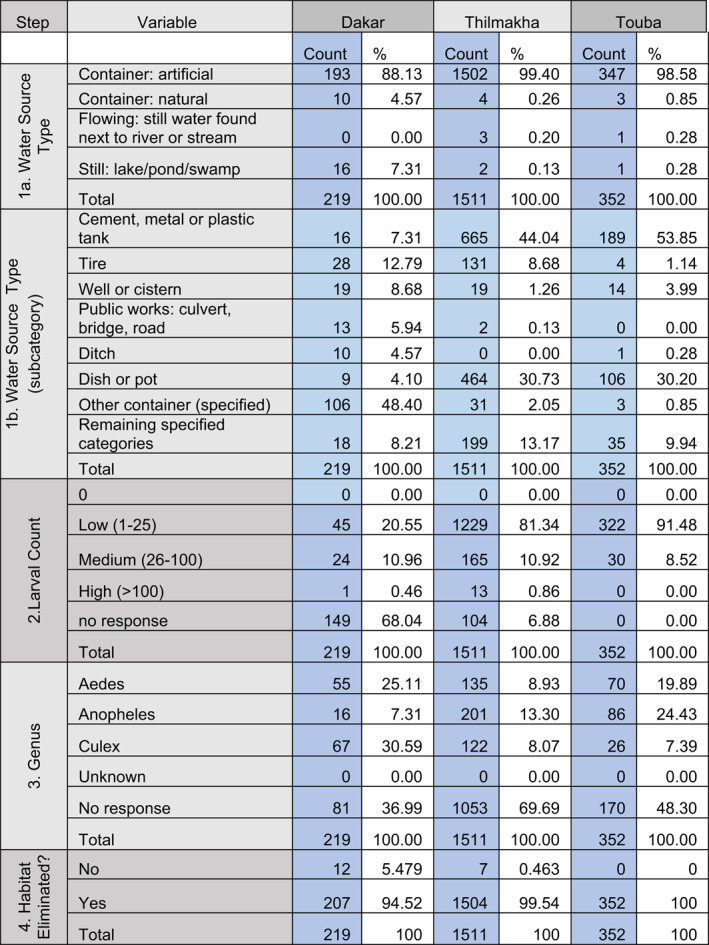

At all three study sites, citizen scientists found most of the larvae in artificial (manufactured) containers (88%–99%). Mosquito larvae were most frequently found in large water containers (cement, metal, or plastic tanks) in Thilmakha (44%) and Touba (54%) In Dakar, abandoned tires (12%), wells and cisterns (9%), and ditch water (5%) were the larval sites where the most mosquito larvae were reported, while outdoor storage tanks were reported far less frequently (7%) than in the non urban regions. The majority of sites in Dakar were reported as “other” (48%), a designation used when the larval habitat does not fit into one of the other 31 categories, and suggests a higher diversity of container habitat types in the complex landscape of a built urban center.

**Figure 3:**
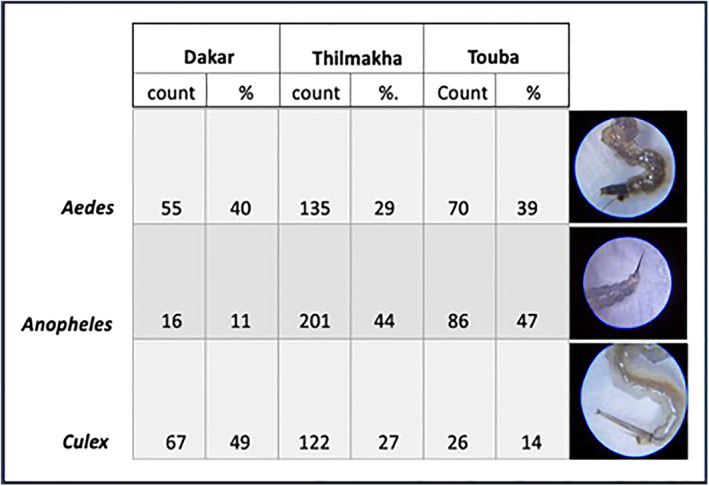
Mosquito genera reported by raw count and percent of total identified specimens for the three study sites. Representative images submitted by Senegalese citizen scientists for each of genera collected by the GLOBE Observer Mosquito Habitat Mapper are pictured on right.

Likewise, Touba and Thilmakha show differences in the reported genera (Figure 3). *Anopheles* mosquitoes were most frequently reported in Touba and Thilmakha, followed by *Aedes*, while citizen scientists in Dakar reported greater numbers of *Aedes* and *Culex*. In the absence of data with meaningful geospatial density, no conclusions can be drawn from the relative numbers or taxa observed between the different communities, but photo documentation supplied with identifications indicate that citizen scientists are capable of providing accurate identifications using the GLOBE Mosquito Habitat Mapper tool.

The number of larval sites eliminated by source reduction by citizen scientists in Senegal is impressively high: Dakar (94%), Thilmakha (99%), and Touba (100%), evidence that this protocol step was stressed in local workshop training sessions. These data also point to the potential for GLOBE Mosquito Habitat Mapper citien science to be a component of an effective vector control strategy when directed by local mosquito control officials.

## 4 Discussion

We have presented the GLOBE Mosquito Habitat Mapper data submitted by citizen scientists over the past three years of the project for three GLOBE Regions: Africa, Asia and the Pacific, and Latin America and the Caribbean. These regions participated in GLOBE's “Engaging Citizens in the Forecasting and Observation of Mosquito Threats” project, which provided training and equipment for target countries beginning in June 2018. After applying quality control routines, which included filtering for spatial errors, and removing records with duplicate or anomalous data, the data set prepared for analysis included records documenting 6485 mosquito larval habitats between May 29, 2017 and May 28, 2020.

The veracity and quality of citizen science data has been questioned by some professional scientists (Aceves‐Bueno et al., 2017; Bonney et al., 2014; Kosmala et al., 2016; Lukyanenko et al., 2016; Nature, 2015). Despite these concerns, citizen science is clearly producing data used in research and policy that is, not available through other means, and has resulted in numerous peer‐reviewed papers (Bonney et al., 2016). From their examination of a range of citizen science projects, De Sherbinin et al. (2020) suggest that the biggest impediment to the use and reuse of citizen science data in scientific research is not the quality of data collected by volunteers, but the lack of detailed documentation that enable scientists to determine whether the data is appropriate and meets the fitness of use requirements of their research design. To this end, GLOBE Observer has published citizen science education and training materials on its website (observer.globe.gov). Metadata records are also available for the GLOBE database (GLOBE, 2019), as well as for this paper (Low et al., 2021). For insight on citizen scientist training and support, workshop presentations and educational materials supporting Mosquito Habitat Mappers are archived on the GLOBE Observer (observer.globe.gov) and GLOBE Mission Mosquito Campaign websites (https://www.globe.gov/web/mission-mosquito).

Data collected opportunistically have unique spatial and temporal biases that pose statistical and data informatics challenges for the end user (Muller et al., 2015). Both the observers and the environmental features or phenomena of interest are not distributed evenly across space and time, and not all observations are equally valuable scientifically (Callaghan et al., 2019). These sampling biases inherent in this data set contrain our ability to make substantive conclusions or comparisons about mosquito populations observed at the global level until a meaningful spatial density of observations populate the database. However, locally collected subsets of Mosquito Habitat Mapper data can provide potentially useful data that can guide community and municipal mosquito control decision making, by providing access to surveillance and mitigation data that is, not duplicated elsewhere.

A comparison of the three community data collection locations in Senegal suggest the value of GLOBE Mosquito Habitat Mapper data on regional, and especially, local spatial scales. There are differences in the use of oviposition sites exhibited between the three locations, as well as the relative percentage of identified taxa. These observations provide opportunities for surveillance teams in these communities to be particularly vigilant when encountering potential oviposition sites and can contribute to the creation of tailored strategies for local scale surveillance and remediation.

The majority of submitted observations did not include identification of the mosquito larva encountered. There may be many reasons contributing to the lack of submissions. Larval identification requires 65–100x magnification. Citizen scientists need to have access to a magnifying device, which may not be available to them locally. The relative difficulty of the larval identification task is high, compared to identifying and documenting larval habitats, and not all citizen scientists will want to spend the time or have sufficient interest to determine the genus or species of their specimen, especially if they do not use the app regularly. The Mosquito Habitat Mapper does not insist on completion of all the steps of the protocol: This trade‐off has been demonstrated to improve citizen science data quality, because volunteers are less inclined to guess at something they do not know in order to complete their observation (Lukyanenko et al., 2019; Torre et al., 2019).

### 4.1 A Tool for Community Health Action: Promoting Source Reduction Behavior

By learning to recognize the varied and sometimes unexpected and cryptic breeding habitats used by mosquitoes, volunteers become knowledgeable about where mosquito larvae are found. Reporting the mitigation of a mosquito larval habitat encourages the healthful practice of eliminating potential standing water breeding sites in and around homes and neighborhoods.

A significant finding in this technical report is the attention citizen scientists paid to source reduction. Source reduction activity was reported almost as frequently as mosquito larval habitats. This data suggests that the citizen scientists using the GLOBE Mosquito Habitat Mapper were highly motivated to reduce the threat of mosquito borne disease in their community. The GLOBE Mosquito Habitat Mapper is one of very few examples of a citizen science reporting tool that collect environmental data alongside behavioral data (Crain et al., 2014). From a human health perspective, the high percentage of larval habitats mitigated by source reduction and reported by participants in the GLOBE Mosquito Habitat Mapper project is a highly significant outcome.

The World Health Organization stresses that source reduction is the most effective intervention for protecting populations (WHO, 2020). However, source reduction is not regularly implemented in many communities. For example, Forsyth et al. (2020) found that the inhabitants of villages in Kenya where they were doing their research were well aware of the importance of bed nets, but they were not well informed about the role of stagnant water in unattended containers in the mosquito lifecycle or the health danger it creates. Forsyth et al. note that source reduction is a health intervention that requires little effort or training, and that even if individuals only target the most productive containers, they can “effectively reduce mosquito indices and relatedly, mosquito‐borne disease risk,” (2020, p. 15). For the many mosquito vector borne diseases for which no commercial vaccine is available, diseases which include malaria, dengue, chikungunya, Zika, West Nile Virus, and Rift Valley fever, adoption of personal protective behaviors is likely the most “promising and effective” way to limit infection (Omodior et al., 2018).

The importance of source reduction to control the spread of mosquito borne disease is echoed in messaging from public health organizations. Warning against the possibility of a deadly spike in dengue fever in Latin America and the Caribbean, the Pan American Health Organization recommends, “For prevention in the home, male and female heads of households are called on to reduce the number of key mosquito breeding sites found in homes,” (PAHO, 2020).

The emergence in Africa of the invasive *Anopheles stephensi* is a game changer with respect to the malaria control landscape (Sinka et al., 2020). *An. stephensi*, unlike many of the other malaria vectors, oviposit in containers similar to *Aedes aegypti* and *Aedes albopictus*. Container breeding mosquitoes are difficult to mitigate, as they opportunistically oviposit in containers of all sizes, from the size of a bottle cap to as large as a cistern or stock tank. Due to challenges in collecting *An. stephensi* adults (Balkew et al., 2020), most efforts for the detection of this invasive species are based on larval surveillance. There is urgent need to limit the spread of *An. stephensi* in Africa and control efforts will rely heavily on early detection of this invasive species in new locations for rapid response. The GLOBE Mosquito Habitat Mapper tool, used by citizen scientists, has utility in *An. stephensi* surveillance efforts that aid in halting the expansion of this invasive malaria vector.

The GLOBE Mosquito Habitat Mapper is a powerful citizen science tool that engages individuals in the three ways society can combat mosquito vector borne diseases for which there is no vaccine: surveillance, mitigation, and education. Citizen science empowers “ordinary people in advancing research endeavors,” (Lukyanenko et al., 2020). By virtue of their personal experience as citizen scientists, participants are better equipped to engage in science‐based public policy discussions using their own authoritative voice.

When deployed by trusted public health partners, a citizen science approach has the potential to be a powerful tool in the face of pandemics, such as the Zika and dengue pandemics that have taken place in South America in recent years. Engagement in citizen science health action can enable individual and “community empowerment by connecting citizens in a common cause, manage misinformation by directly engaging citizens, and inform evidence‐based decision making using big data,” (Katapally, 2020).

Citizen science has been called out as a transformative methodology to address and monitor not only health, but all of the United Nations Sustainable Development Goals (Balázs et al., 2021; Fritz et al., 2019). The GLOBE Mosquito Habitat Mapper is a no‐cost mobile app tool that is, available for rapid emergency deployment by public health and mosquito control agencies in response to extreme weather events and mosquito vector borne disease outbreaks. It is our hope that GLOBE Mosquito Habitat Mapper will foster the “democratization of science and the mobilization of diverse people and communities,” (EPA, 2016, p. 45), and empower the public to take action and play a role in protecting the health of themselves, their families and their neighborhoods.

## 5 Conclusions

The GLOBE Mosquito Habitat Mapper is a research tool which can be used in systematic research at many different scales. It is available in 12 languages and in 124 countries, enabling citizen scientists to be activated in rapid response to real‐time mosquito borne health threats posed in the aftermath of extreme weather events or during a pandemic.

The global data set is collected using a common data collection protocol and tool, creating consistency in variables and attributes reported to the GLOBE database. These data can be further used in a data driven approach by scientists who may not have been part of the original data collection. With increased and more widespread adoption of the GLOBE Mosquito Habitat Mapper by citizen scientists, there will be expanded opportunities for scientific discovery on multiple scales.

When the GLOBE Mosquito Habitat Mapper is deployed within a community to address a locally relevant issue, project leaders can choose the steps that provide the specific data they need. This information design feature supports increased data quality, but it also makes the GLOBE Mosquito Habitat Mapper a versatile data collection tool that is, appropriate for understanding a variety of research questions. While the GLOBE Mosquito Habitat Mapper data as a whole is subject to the analytic limitations of opportunistically collected data, local project leads are free to identify and instruct the public how to collect data so that the data is obtained using a systematic spatial or temporal sampling design or standardized volume sampling. The team function in app enables data obtained using a specific sampling protocol to be associated digitally for future analysis.

The GLOBE Mosquito Habitat Mapper protocol is a relatively recent addition to GLOBE. The intended purpose of this citizen science tool is to provide ground reference data to support the analysis of digital data obtained from remote sensing platforms, still awaits the future accumulation of data at a meaningful geospatial density to fully realize this task. Until then, the GLOBE Observer Mosquito Habitat Mapper will continue to support the efforts of local mosquito control agencies and citizen scientists who want to be agents of change and reduce the risk of mosquito‐borne disease in their own communities.

## Conflict of Interest

The authors declare no conflicts of interests relevant to this study.

## Data Availability

GLOBE data are publicly available at https://www.globe.gov/globe-data. These data were obtained from NASA and the GLOBE Program and are freely available for use in research, publications and commercial applications. When data from GLOBE are used in a publication, we request this acknowledgment be included: "These data were obtained from the GLOBE Program." Please include such statements, either where the use of the data or other resource is described, or within the Acknowledgements section of the publication. Curated data sets on which this article is based, as well as the Python code employed in quality assurance and metadata descriptions are available at https://geospatial.strategies.org/pages/publication-data. Data citation: Low, R., Boger, B., Nelson, P. and Kimura, M (2021). GLOBE Observer Mosquito Habitat Mapper Citizen Science Data 2017–2020, v1.0. (https://doi.org/10.5281/zenodo.5106570).
